# Low-Altitude Windshear Wind Speed Estimation Method Based on KASPICE-STAP

**DOI:** 10.3390/s23010054

**Published:** 2022-12-21

**Authors:** Hai Li, Yutong Chen, Kaihong Feng, Ming Jin

**Affiliations:** 1 Tianjin Key Lab for Advanced Signal Processing, Civil Aviation University of China, Tianjin 300300, China; 2 Faculty of Electrical Engineering and Computer Science, Ningbo University, Ningbo 315211, China

**Keywords:** airborne weather radar, clutter suppression, KASPICE-STAP, SPICE algorithm, wind speed estimation

## Abstract

Aiming at the problem of low-altitude windshear wind speed estimation for airborne weather radar without independent identically distributed (IID) training samples, this paper proposes a low-altitude windshear wind speed estimation method based on knowledge-aided sparse iterative covariance-based estimation STAP (KASPICE-STAP). Firstly, a clutter dictionary composed of clutter space–time steering vectors is constructed using prior knowledge of the distribution position of ground clutter echo signals in the space–time spectrum. Secondly, the SPICE algorithm is used to obtain the clutter covariance matrix iteratively. Finally, the STAP processor is designed to eliminate the ground clutter echo signal, and the wind speed is estimated after eliminating the ground clutter echo signal. The simulation results show that the proposed method can accurately realize a low-altitude windshear wind speed estimation without IID training samples.

## 1. Introduction

Low-altitude windshear is an atmospheric phenomenon in which the air flow below 600 m suddenly changes its direction or speed within a small area. It has the characteristics of a short duration, a small area of action, a high instantaneous intensity, and a strong potential to cause harm [1]. When an aircraft suddenly encounters low-altitude windshear in the takeoff or landing stage, the pilot usually lacks sufficient time and space to control the attitude of the aircraft, which leads to the occurrence of flight accidents. Therefore, the detection and warning of low-altitude windshear has become an important topic in the current civil aviation field [2]. The estimation of low-altitude windshear wind speed determines the accuracy of low-altitude windshear detection, so the accurate estimation of low-altitude windshear wind speed is of much concern. As airborne weather radar operates in look-down mode, the clutter has a wide distribution range and a high intensity, and the aircraft’s movement will cause the clutter spectrum to expand seriously, which will lead to the low-altitude windshear echo signal in the airborne weather radar echo signal often being submerged in the ground clutter echo signal, so the suppression effect of the ground clutter echo signal will directly affect the accuracy of the wind speed estimation [3].

Space-–time adaptive processing (STAP) technology [4] is a signal-processing technology that is applied to airborne weather radar. The application of STAP technology makes it possible to accurately estimate the low-altitude windshear wind speed in strong clutter environments. The optimal weight vector of STAP relies on the accurate estimation of the clutter covariance matrix (CCM) [5]. According to the RMB (Reed–Mallet–Brennan) criterion proposed by Reed et al. [6], the used training samples must be independent identically distributed (IID) samples, and only when the number of IID training samples reaches two times the freedom degree of the system or more can the performance of the traditional STAP method be reduced by less than 3 dB. However, in practice, the lack of IID training samples makes the traditional STAP technology unable to accurately obtain the clutter covariance matrix, which leads to the degradation of the ground clutter echo signal suppression performance, and this limits the application of the traditional STAP technology in low-altitude windshear wind-speed estimation. Although the rank-reduction STAP [7] and dimensionality-reduction STAP [8], which were proposed to estimate the low-altitude wind speed of windshear when the number of IID training samples is low, can effectively suppress the ground clutter echo signal and estimate the wind speed, they still need a certain number of IID training samples.

In recent years, scholars have proposed a variety of STAP methods that do not require IID training samples. In 2009, Sarkar et al. [9] proposed the direct data domain STAP (DDD-STAP) method. This method directly determines the window for the detection range units without IID training samples, which can effectively suppress clutter and achieve the purpose of target detection. However, the sliding window involve the loss of the freedom degree of the system, and the sliding window has high requirements on the degrees of freedom in the space and time domains. The development of knowledge-aided STAP (KA-STAP) technology brings a new round of innovation to STAP technology. Although KA-STAP technology can improve the clutter suppression performance to a certain extent, it requires a higher precision of prior knowledge. On this basis, in 2011, Sun Ke et al. from Tsinghua University introduced a direct data domain and prior knowledge into the sparse STAP method and proposed the DDD sparse recovery STAP (DDD-SR-STAP) method [10]. In this method, the detection range units were directly sparse recovered without a sliding window, but this requires prior knowledge of the specific position distribution of the echo signal in the target area in the two-dimensional space–time spectrum. Since the low-altitude windshear is a distributed target and the low-altitude windshear has the characteristics of a sudden occurrence and a short existence time, it is very difficult to know the specific position distribution of low-altitude windshear echo signals in a two-dimensional space–time spectrum in advance.

STAP methods that require few or no IID samples have been applied in clutter suppression and parameter estimation in many ways [11,12,13,14,15,16,17,18], but they are all aimed at point targets and cannot be directly applied to distributed targets such as low-altitude windshear. Therefore, it is of great significance to study how to accurately realize low-altitude windshear wind speed estimation without IID training samples.

Aiming at solving the above problems, this paper proposes a low-altitude windshear wind speed estimation method based on knowledge-aided sparse iterative covariance-based estimation STAP (KASPICE-STAP). Firstly, a clutter dictionary composed of clutter space–time steering vectors is constructed by using prior knowledge of the distribution position of the space–time spectrum of the ground clutter echo signals. Secondly, the SPICE algorithm is used to obtain the clutter covariance matrix iteratively. Finally, the STAP processor is designed to calculate the corresponding optimal weight vector for clutter suppression, the normalized Doppler frequency is obtained from the signal after clutter suppression, and then, the wind speed is estimated. Simulation results show that the proposed method can effectively and accurately estimate wind speed without IID training samples.

## 2. Echo Signal Model

**Figure 1:**
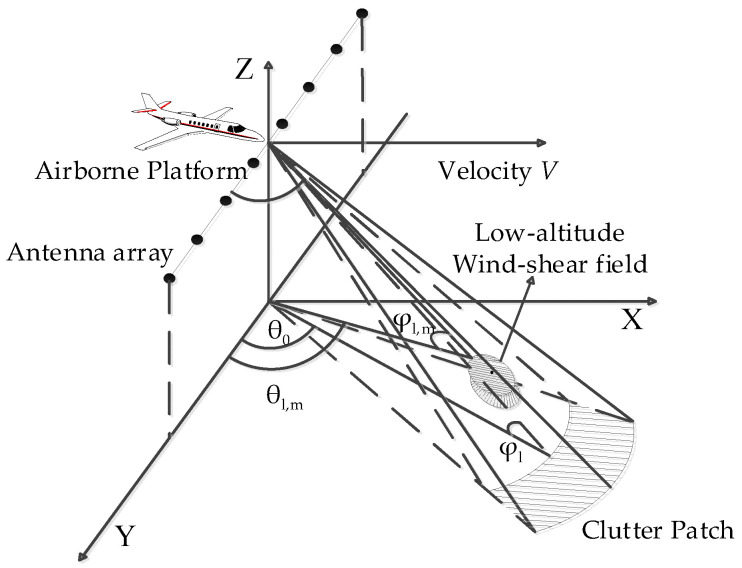
The model of low-altitude windshear detected by an airborne weather radar forward-looking array.

The model of low-altitude windshear detected by an airborne weather radar forward-looking array is shown in Figure 1. The reference coordinate system is established with H meters directly below the particle point of the aircraft platform as the origin. It is assumed that the antenna array is composed of an N-element linear array, and it is placed evenly along the Y-axis with a spacing of d=0.5 λ, where λ is the wavelength. We assume that the pulse repetition rate is $f_{r}$ and the number of pulses within a coherent processing interval is K. In Figure 1, $\theta_{0}$ is the horizontal azimuth angle of low-altitude windshear wind field echo signal, $\varphi_{l}$ is its pitch angle, $\psi_{0}$ is its spatial cone angle, and $\cos\psi_{0}=\cos\varphi_{l}\cos\theta_{0}$ is valid. 

This paper assumes that there are L range units in total; $x_{l}$ represents the NK×1-dimensional radar echo signal of the lth range unit to be detected (l=1,2,……,L), and its expression is:

(1) $$x_{l}=s_{l}+c_{l}+N_{l}$$

where, $s_{l}$ is the low-altitude windshear wind field echo signal in the lth range unit to be detected; $c_{l}$ is the ground clutter echo signal in the lth range unit to be detected; $n_{l}$ is additive white Gaussian noise.

### 2.1. Low-Altitude Windshear Echo Signal Model

The echo signal $s_{l}$ of low-altitude windshear wind field in the lth range unit to be detected is:

(2) $$s_{l}=\sigma .v_{l}{\left(f_{d},\psi_{0}\right)}_{NK\times 1}=\sigma .v_{d}{\left(f_{d}\right)}_{K\times 1}⊗v_{s}{\left(\psi_{0}\right)}_{N\times 1}$$

where ⊗ is the Kronecker product; σ is the complex amplitude of the low-altitude windshear wind field echo signal of the lth range unit to be detected; $\psi_{0}$ is the spatial cone angle of low-altitude windshear wind field echo signal. In this paper, it is considered that the spatial cone angle of low-altitude windshear wind field echo signal is prior information. $f_{d}$ is the normalized Doppler frequency of the wind field echo signal in the lth range unit to be detected, and the value range is [−1,1]; $v_{l}{\left(f_{d},\psi_{0}\right)}_{NK\times 1}$ is the space–time steering vector of wind field echo signal in the lth range unit to be detected; $v_{d}{\left(f_{d}\right)}_{K\times 1}$ and $v_{s}{\left(\psi_{0}\right)}_{N\times 1}$ are, respectively, the time steering vector and space steering vector, constituting the space–time steering vector of the wind field echo signal in the range unit to be detected, and the expressions are as follows:

(3) $$v_{d}{\left(f_{d}\right)}_{K\times 1}={\left[1,e^{J\pi f_{d}},\ldots \ldots ,e^{J\pi \left(K-1\right)f_{d}}\right]}_{K\times 1}^{T}⊙g(\sigma_{f_{d}})$$

(4) $$\begin{array}{c} v_{s}{\left(\psi_{0}\right)}_{N\times 1}={\left[1,e^{J2\pi \frac{d}{\lambda }\cos\theta_{0}\cos\varphi_{l}},\ldots \ldots ,e^{J2\pi \left(N-1\right)\frac{d}{\lambda }\cos\theta_{0}\cos\varphi_{l}}\right]}_{N\times 1}^{T}⊙g(\sigma_{\psi_{0}})=\\ {\left[1,e^{J2\pi \frac{d}{\lambda }\cos\psi_{0}},\ldots \ldots ,e^{J2\pi \left(N-1\right)\frac{d}{\lambda }\cos\psi_{0}}\right]}_{N\times 1}^{T}⊙g(\sigma_{\psi_{0}}) \end{array}$$

In order to accurately describe the distributed model such as low-altitude windshear, Meng et al. [19] gradually established and improved the spatial distribution source model. By introducing an extension function to describe the angle and frequency expansion of the signal, the model extends the space–time steering vector of the point target to obtain the space–time steering vector of the distributed target. In Equations (3) and (4), ⊙ is the Hadamard product; $g(\sigma_{f_{d}})={\left[1,e^{-2\pi^{2}\sigma_{f_{d}}},\ldots \ldots ,e^{-2\pi^{2}(K-1)\sigma_{f_{d}}}\right]}^{T}$ is the frequency expansion function of the low-altitude windshear wind field echo signal in the range unit to be detected; $g(\sigma_{\psi_{0}})={\left[1,e^{-1/2{\left(2\pi \frac{d}{\lambda }\right)}^{2}\sigma_{\psi_{0}}},\ldots \ldots ,e^{-1/2{\left(2\pi (N-1)\frac{d}{\lambda }\right)}^{2}\sigma_{\psi_{0}}}\right]}^{T}$ is the angle expansion function of the low-altitude windshear wind field echo signal in the range unit to be detected;$\sigma_{\psi_{0}}=\sigma_{\theta_{0}}^{2}\cos^{2}\varphi_{l}\sin^{2}\theta_{0}+\sigma_{\varphi_{l}}^{2}\sin^{2}\varphi_{l}\cos^{2}\theta_{0}$, $\sigma_{\theta_{0}}$ represents the extension of $\theta_{0}$ in the horizontal azimuth angle of the low-altitude windshear wind field echo signal; $\sigma_{\varphi_{l}}$ represents the extension of $\varphi_{l}$ in the direction of its pitch angle [20].

### 2.2. Ground Clutter Echo Signal Model

The ground clutter echo signal model of the airborne weather radar in this paper is established based on the Ward model [21] which was developed by Lincoln Laboratory of Massachusetts Institute of Technology. The ground clutter in the lth range unit to be detected is divided into M ground clutter patches, the mth ground clutter patch is shown in Figure 1. It is assumed that the ground clutter echo signal has no fluctuation and no fuzzy phenomenon [22], and the ground clutter echo signal $c_{l}$ in the lth range unit to be detected is:

(5) $$c_{l}=\sum_{m=1}^{m}\frac{B_{m}}{R_{l}^{2}}f\left(\theta_{l,m}\right)a(f_{dm},\psi_{sm})$$

where $B_{m}$ is the reflection coefficient of the mth ground clutter patch; $F\left(\theta_{l,m}\right)$ is the radar antenna pattern; $r_{l}$ is the distance between the ground clutter patches of the lth range unit to be detected and the aircraft platform; $a(f_{d,m},\psi_{s,m})$ is the NK×1-dimensional space–time steering vector of the mth ground clutter patch. The expression is as follows:

(6) $$a(f_{\mathrm{dm}},\psi_{\mathrm{sm}})={\left[1,e^{J\pi f_{dm}},\ldots \ldots ,e^{J\pi (K-1)f_{dm}}\right]}_{K\times 1}^{T}⊗{\left[1,e^{J2\pi \frac{d}{\lambda }\cos\psi_{sm}},\ldots \ldots ,e^{J2\pi (N-1)\frac{d}{\lambda }\cos\psi_{sm}}\right]}_{N\times 1}^{T}$$

where $\psi_{\mathrm{sm}}$ is the corresponding spatial cone angle; $f_{\mathrm{dm}}$ is the normalized Doppler frequency; when the pitch angle and horizontal azimuth angle of the clutter patch in this area are $\varphi_{l,m}$ and $\theta_{l,m}$, respectively, then the spatial cone angle and the normalized Doppler frequency can be expressed as:

(7) $$\psi_{s,m}=\arccos(\cos\theta_{l,m}\cos\varphi_{l,c})$$

(8) $$f_{d,m}=\frac{4V}{\lambda f_{r}}\cos(\alpha -\theta_{l,m})\cos\varphi_{l,c}$$

where α is the included angle between the flight direction of the aircraft and the antenna array, and it is 90°.

## 3. Low-Altitude Windshear Wind Speed Estimation Using KASPICE-STAP Method

The estimation method of low-altitude windshear wind speed based on KASPICE-STAP is mainly divided into two parts: a clutter covariance matrix estimation based on SPICE algorithm and a low-altitude windshear wind speed estimation. The following are discussed, respectively.

### 3.1. Estimation of Clutter Covariance Matrix Based on SPICE Algorithm

To estimate the clutter covariance matrix by the SPICE algorithm, first of all, the clutter dictionary is constructed by using prior knowledge of the distribution position of the ground clutter echo signals in space–time two-dimensional spectrum, and then, the clutter covariance matrix is estimated by the SPICE algorithm. In the following two aspects, the construction of the clutter dictionary and the estimation of the clutter covariance matrix by the SPICE algorithm are described.

#### 3.1.1. Construction of Clutter Dictionary

**Figure 2:**
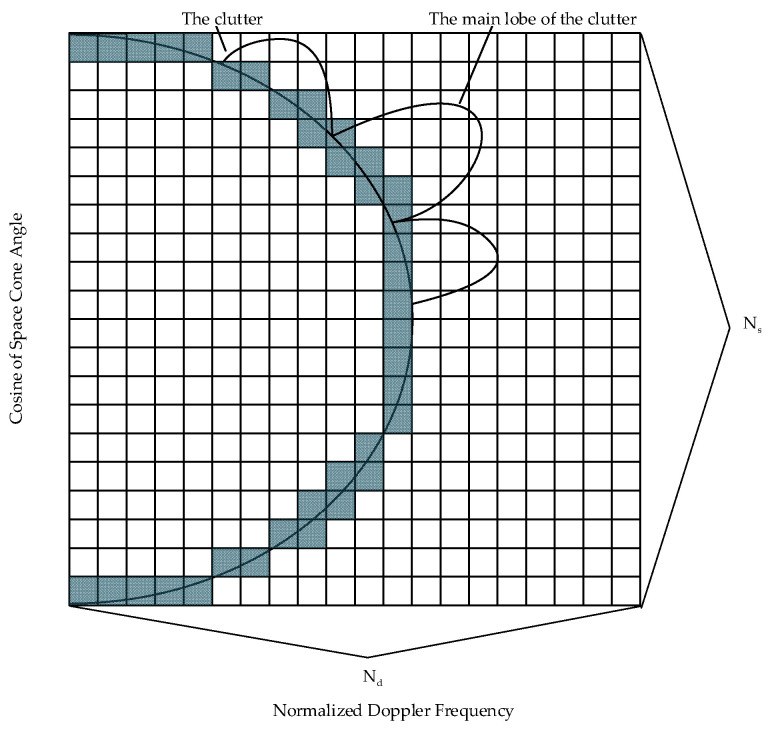
Location distribution of ground clutter in space–time two-dimensional spectrum.

The position distribution of the ground clutter in space–time two-dimensional spectrum is shown in Figure 2. In the figure, the gray cell grids are the distribution position of the ground clutter echo signals, and the white cell grids are the distribution position of noise and low-altitude windshear echo signals. Since it is impossible to divide the clutter infinitely as in practical applications, a discrete approach is adopted to approximate the division, and each clutter cell grid is evenly divided into $N_{s}$ parts. Then, the NK×1-dimensional space–time steering vector corresponding to each clutter cell grid is $a(f_{\mathrm{dm}},\psi_{\mathrm{sm}})=s_{\mathrm{dm}}(f_{\mathrm{dm}})⊗s_{\mathrm{sm}}(\psi_{\mathrm{sm}}),m=1,2,\ldots ,N_{s}$, where $s_{\mathrm{dm}}(f_{\mathrm{dm}})=[1,e^{J\pi f_{\mathrm{dm}}},\ldots ,e^{J\pi (K-1)f_{\mathrm{dm}}}]$ is the time steering vector and $s_{\mathrm{sm}}(\psi_{\mathrm{sm}})=[1,e^{J2\pi \frac{d}{\lambda }\psi_{\mathrm{dm}}},\ldots ,e^{J2\pi (N-1)\frac{d}{\lambda }\psi_{\mathrm{dm}}}]$ is the space steering vector. Then, the space–time steering vector of each clutter cell grid is combined into an $NK\times N_{s}$ dimensional clutter dictionary $A_{1}$, which is expressed as:

(9) $$A_{1}=[a(f_{d1},\psi_{s1}),\ldots ,a(f_{\mathrm{dm}},\psi_{\mathrm{sm}}),\ldots ,a(f_{dN_{s}},\psi_{sN_{s}})]$$

#### 3.1.2. Estimation of Clutter Covariance Matrix Using SPICE Algorithm

If we let $R_{l}$ be the clutter covariance matrix of the lth range unit that is to be detected, its expression is [23]:

(10) $$R_{l}=E\left[\left(c_{l}+n_{l}\right){\left(c_{l}+n_{l}\right)}^{H}\right]$$

As it can be seen from Figure 2, since a single clutter cell grid is evenly divided into $N_{s}$ parts, M in Equation (5) is $N_{s}$, and $\alpha_{l,m}=\frac{B_{m}}{R_{l}^{2}}F\left(\theta_{l,m}\right)$ is the amplitude value of the ground clutter echo signal of the lth range unit that is to be detected and the mth clutter cell grid. In this case, $c_{l}$ in Equation (5) can be expressed as:

(11) $$c_{l}=\sum_{m=1}^{N_{s}}\alpha_{l,m}.a(f_{\mathrm{dm}},\psi_{\mathrm{sm}})$$

By substituting Equation (11) into Equation (10), we can obtain:

(12) $$\begin{array}{l} R_{l}=E\left[\left(c_{l}+n_{l}\right){\left(c_{l}+n_{l}\right)}^{H}\right]\\ =E[\left(\sum_{m=1}^{N_{s}}\alpha_{l.m}.a(f_{\mathrm{dm}},\psi_{\mathrm{sm}})+n_{l}\right){\left(\sum_{m=1}^{N_{s}}\alpha_{l,m}.a(f_{dm},\psi_{\mathrm{sm}})+n_{l}\right)}^{H})]\\ =E\left[\sum_{m=1}^{N_{s}}{\left|\alpha_{l,m}\right|}^{2}a(f_{\mathrm{dm}},\psi_{\mathrm{sm}}){\left(a(f_{\mathrm{dm}},\psi_{\mathrm{sm}})\right)}^{H}\right]\\ +E\left[n_{l}^{H}\sum_{m=1}^{N_{s}}\alpha_{l,m}.a(f_{\mathrm{dm}},\psi_{\mathrm{sm}})\right]+E\left[n_{l}\sum_{m=1}^{N_{s}}\alpha_{l,m}.{\left(a(f_{\mathrm{dm}},\psi_{\mathrm{sm}})\right)}^{H}\right]+E\left[n_{l}n_{l}^{H}\right] \end{array}$$

It is assumed that since clutter and noise are independent of each other [24], $E\left[n_{l}^{H}\sum_{m=1}^{N_{s}}\alpha_{l,m}.a(f_{\mathrm{dm}},\psi_{\mathrm{sm}})\right]=0$, $E\left[n_{l}\sum_{m=1}^{N_{s}}\alpha_{l,m}.{\left(a(f_{\mathrm{dm}},\psi_{\mathrm{sm}})\right)}^{H}\right]=0$ in Equation (12) can be obtained by substituting it into Equation (12):

(13) $$R_{l}=E\left[\sum_{m=1}^{N_{s}}{\left|\alpha_{l,m}\right|}^{2}a(f_{\mathrm{dm}},\psi_{\mathrm{sm}}){\left(a(f_{\mathrm{dm}},\psi_{\mathrm{sm}})\right)}^{H}\right]+E\left[n_{l}n_{l}^{H}\right]$$

In Equation (13), $E\left[n_{l}n_{l}^{H}\right]=\left[\begin{array}{cccc} \sigma_{1} & 0 & \cdots & 0\\ 0 & \sigma_{2} & \cdots & 0\\ \vdots & \vdots & \ddots & \vdots \\ 0 & \cdots & \cdots & \sigma_{n} \end{array}\right]=\sigma_{N}I_{NK\times NK}$. Therefore, Equation (13) can be expressed as:

(14) $$R_{l}=\sum_{m=1}^{N_{s}}{\left|\alpha_{l,m}\right|}^{2}a(f_{\mathrm{dm}},\psi_{\mathrm{sm}}){\left(a(f_{\mathrm{dm}},\psi_{\mathrm{sm}})\right)}^{H}+\sigma_{n}I_{NK\times NK}$$

where ${\left|\alpha_{l,m}\right|}^{2}$ is called the sparse coefficient vector, which represents the power value of the ground clutter echo signal of the lth range unit that is to be detected and the mth clutter cell grid. $\sigma_{N}$ is the receiver noise power, which is regarded as a known quantity.

In this case, the clutter covariance matrix can be estimated through Equation (14). In Equation (14), ${\left|\alpha_{l,m}\right|}^{2}$ is an unknown quantity, while in this paper, to solve the value of ${\left|\alpha_{l,m}\right|}^{2}$, the SPICE algorithm is used, and its objective function and constraint condition are as follows:

(15) $$\{\begin{array}{c} \min\;J={\left\|\left(\sum_{m=1}^{N_{s}}{\left|\alpha_{l,m}\right|}^{2}a(f_{\mathrm{dm}},\psi_{sm}){\left(a(f_{\mathrm{dm}},\psi_{sm})\right)}^{H}+\sigma_{n}I_{NK\times NK})({\overset{⌢}{R}}_{l}-(\sum_{m=1}^{N_{s}}{\left|\alpha_{l,m}\right|}^{2}a(f_{\mathrm{dm}},\psi_{sm}){\left(a(f_{\mathrm{dm}},\psi_{\mathrm{sm}})\right)}^{H}+\sigma_{n}I_{NK\times NK})\right)\right\|}_{F}^{2}\\ s.t.\frac{{\left\|{\left(\mathrm{diag}({\left|\alpha_{l,m}\right|}^{2})\right)}^{t+1}-{\left(\mathrm{diag}({\left|\alpha_{l,m}\right|}^{2})\right)}^{t}\right\|}_{2}^{2}}{{\left\|{\left(\mathrm{diag}({\left|\alpha_{l,m}\right|}^{2})\right)}^{t}\right\|}_{2}^{2}}\leq \kappa \end{array}$$

In Equation (15), J is the objective function of the SPICE algorithm, which is to obtain the value of ${\left|\alpha_{l,m}\right|}^{2}$ by solving the minimum value problem of J and judging whether the constraint condition is valid. If the answer is yes, then ${\left|\alpha_{l,m}\right|}^{2}$ is the desired value; otherwise, we keep iterating until the value of ${\left|\alpha_{l,m}\right|}^{2}$ meets the constraint condition. ${\overset{⌢}{R}}_{l}$ is the maximum likelihood estimate of the covariance matrix; $\mathrm{diag}({\left|\alpha_{l,m}\right|}^{2})$ is the power diagonal matrix of the clutter space–time two-dimensional spectrum formed by the sparse coefficient vector ${\left|\alpha_{l,m}\right|}^{2}$; κ represents the threshold of SPICE algorithm accuracy level, generally, the empirical value is 0.13; t+1 is the current number of times that we need to use the SPICE algorithm, t=0,1,…,N. For convenient representation, we substitute Equation (14) into the expression of J in Equation (15) and mark $\mathrm{diag}({\left|\alpha_{l,m}\right|}^{2})$ as B. Equation (15) can be expressed as:

(16) $$\left\{\begin{array}{c} J={\left\|R_{l}^{-\frac{1}{2}}({\overset{⌢}{R}}_{l}-R_{l})\right\|}_{F}^{2}\\ s.t.\frac{{\left\|B^{t+1}-B^{t}\right\|}_{2}^{2}}{{\left\|B^{t}\right\|}_{2}^{2}}\leq \kappa \end{array}\right.$$

Since the proposed method does not use IID training samples, as it only uses the range unit $x_{l}$ to be detected, the ${\overset{⌢}{r}}_{l}$ expression is as follows:

(17) $${\overset{⌢}{r}}_{l}=x_{l}x_{l}^{H}$$

We substitute Equation (17) into the objective function J expression of Equation (16) and expand and simplify the F norm to obtain:

(18) $$\begin{array}{cc} J & ={\left\|R_{l}^{-\frac{1}{2}}(x_{l}x_{l}^{H}-R_{l})\right\|}_{F}^{2}\\ & =Tr\left[{\left(x_{l}x_{l}^{H}-R_{l}\right)}^{H}{\left(R_{l}^{-\frac{1}{2}}\right)}^{H}R_{l}^{-\frac{1}{2}}(x_{l}x_{l}^{H}-R_{l})\right]\\ & =Tr\left[R_{l}^{-1}{\left(x_{l}x_{l}^{H}-R_{l}\right)}^{2}\right]\\ & =Tr(x_{l}x_{l}^{H})x_{l}^{H}R_{l}^{-1}x_{l}+Tr(R_{l})-2{\left\|x_{l}\right\|}_{2}^{2}\\ & ={\left\|x_{l}\right\|}_{2}^{2}x_{l}^{H}R_{l}^{-1}x_{l}+E\left[{\left\|x_{l}\right\|}_{2}^{2}\right]-2{\left\|x_{l}\right\|}_{2}^{2} \end{array}$$

Strictly speaking, the mathematical expectation $E\left[{\left\|x_{l}\right\|}_{2}^{2}\right]$ of L2-norm square of $x_{l}$ cannot be solved exactly [25]. However, since the radar echo signal $x_{l}$ of the lth range unit to be detected is superimposed by the ground clutter echo signal, noise, and low-altitude windshear echo signal, it can be written as follows:

(19) $$E\left[{\left\|x_{l}\right\|}_{2}^{2}\right]=E\left[{\left\|c_{l}+n_{l}+s_{l}\right\|}_{2}^{2}\right]$$

Therefore, Equation (11) is substituted into Equation (19), and according to literature [24], we can obtain:

(20) $$\begin{array}{ll} E\left[{\left\|x_{l}\right\|}_{2}^{2}\right] & =E\left[{\left\|c_{l}+n_{l}+s_{l}\right\|}_{2}^{2}\right]=E\left[{\left\|\sum_{m=1}^{N_{s}}\alpha_{l,m}.a(f_{\mathrm{dm}},\psi_{\mathrm{sm}})+n_{l}+s_{l}\right\|}_{2}^{2}\right]\\ & =\sum_{m=1}^{N_{s}}{\left|\alpha_{l,m}\right|}^{2}{\left\|a(f_{\mathrm{dm}},\psi_{\mathrm{sm}})\right\|}_{2}^{2}+NK\sigma_{n}+{\left\|s_{l}\right\|}_{2}^{2} \end{array}$$

Substituting Equation (20) into Equation (18) to obtain:

(21) $$J={\left\|x_{l}\right\|}_{2}^{2}x_{l}^{H}R_{l}^{-1}x_{l}+\sum_{m=1}^{N_{s}}{\left|\alpha_{l,m}\right|}^{2}{\left\|a(f_{\mathrm{dm}},\psi_{\mathrm{sm}})\right\|}_{2}^{2}+NK\sigma_{n}+{\left\|s_{l}\right\|}_{2}^{2}-2{\left\|x_{l}\right\|}_{2}^{2}$$

The last two terms in Equation (21) are constants, which do not affect the result of solving its minimum value problem, so after ignoring the ${\left\|s_{l}\right\|}_{2}^{2}-2{\left\|x_{l}\right\|}_{2}^{2}$, the expression for $J_{1}$ is:

(22) $$J_{1}={\left\|x_{l}\right\|}_{2}^{2}x_{l}^{H}R_{l}^{-1}x_{l}+\sum_{m=1}^{N_{s}}{\left|\alpha_{l,m}\right|}^{2}{\left\|a(f_{\mathrm{dm}},\psi_{\mathrm{sm}})\right\|}_{2}^{2}+NK\sigma_{n}$$

Then, the ${\left|\alpha_{l,m}\right|}^{2}$ solution obtained by solving the minimum problem of J is the same as that obtained by solving the minimum problem of $J_{1}$. Since ${\left\|x_{l}\right\|}_{2}^{2}$ is a constant, to simplify the calculation, we divide both of the sides of Equation (22) by ${\left\|x_{l}\right\|}_{2}^{2}$ at the same time to obtain:

(23) $$\frac{J_{1}}{{\left\|x_{l}\right\|}_{2}^{2}}=x_{l}^{H}R_{l}^{-1}x_{l}+\sum_{m=1}^{N_{s}}{\left|\alpha_{l,m}\right|}^{2}\frac{{\left\|a(f_{\mathrm{dm}},\psi_{\mathrm{sm}})\right\|}_{2}^{2}}{{\left\|x_{l}\right\|}_{2}^{2}}+NK\frac{\sigma_{n}}{{\left\|x_{l}\right\|}_{2}^{2}}$$

Let the expression $J_{2}$ be:

(24) $$J_{2}=x_{l}^{H}R_{l}^{-1}x_{l}+\sum_{m=1}^{N_{s}}{\left|\alpha_{l,m}\right|}^{2}\frac{{\left\|a(f_{\mathrm{dm}},\psi_{\mathrm{sm}})\right\|}_{2}^{2}}{{\left\|x_{l}\right\|}_{2}^{2}}+NK\frac{\sigma_{n}}{{\left\|x_{l}\right\|}_{2}^{2}}$$

Then, solving the minimum problem of $J_{1}$ has the same solution as solving the minimum problem of $J_{2}$. According to the study of Rojas et al. [26], the minimum problem of Equation (24) can be written as:

(25) $$\left\{\begin{array}{l} \min x_{l}^{H}R_{l}^{-1}x_{l}\\ s.t.\sum_{m=1}^{N_{s}}{\left|\alpha_{l,m}\right|}^{2}\frac{{\left\|a(f_{\mathrm{dm}},\psi_{\mathrm{sm}})\right\|}_{2}^{2}}{{\left\|x_{l}\right\|}_{2}^{2}}+NK\frac{\sigma_{n}}{{\left\|x_{l}\right\|}_{2}^{2}}\leq 1 \end{array}\right.$$

According to the literature [23], to solve Equation (25), the clutter covariance matrix $R_{l}$ in Equation (25) is expressed as:

(26) $$R_{l}^{}=\Theta_{}^{H}B\Theta$$

where $\Theta =[A_{1},I_{NK}]$ is a $NK\times (N_{s}+NK)$-dimensional sparse dictionary composed of clutter and noise. By substituting Equation (26) into Equation (25), we can obtain:

(27) $$\left\{\begin{array}{l} \min x_{l}^{H}{\left(\Theta_{}^{H}B\Theta \right)}^{-1}x_{l}\\ s.t.\sum_{m=1}^{N_{s}}{\left|\alpha_{l,m}\right|}^{2}\frac{{\left\|a(f_{\mathrm{dm}},\psi_{\mathrm{sm}})\right\|}_{2}^{2}}{{\left\|x_{l}\right\|}_{2}^{2}}+NK\frac{\sigma_{n}}{{\left\|x_{l}\right\|}_{2}^{2}}\leq 1 \end{array}\right.$$

In this case, in order to solve the minimum problem of Equation (27), a new matrix Ψ is introduced, which satisfies:

(28) $$\Psi ={\left(\Theta^{-1}\right)}^{H}$$

Namely:

(29) $$\left\{\begin{array}{l} \Theta ={\left(\Psi^{H}\right)}^{-1}\\ \Psi^{H}\Theta =I_{NK} \end{array}\right.$$

By substituting Equation (29) into Equation (27), we can obtain:

(30) $$\left\{\begin{array}{l} \min x_{l}^{H}{\left(\Theta_{}^{H}B\Theta \right)}^{-1}x_{l}=\min x_{l}^{H}\Theta^{-1}B^{-1}{\left(\Theta^{H}\right)}^{-1}x_{l}=\min x_{l}^{H}\Psi^{H}B^{-1}\Psi x_{l}\\ s.t.\Psi^{H}\Theta =I_{NK}\\ \sum_{m=1}^{N_{s}}{\left|\alpha_{l,m}\right|}^{2}\frac{{\left\|a(f_{\mathrm{dm}},\psi_{\mathrm{sm}})\right\|}_{2}^{2}}{{\left\|x_{l}\right\|}_{2}^{2}}+NK\frac{\sigma_{n}}{{\left\|x_{l}\right\|}_{2}^{2}}\leq 1 \end{array}\right.$$

According to the literature [27,28,29], the solution to the minimum value problem is:

(31) $$\left\{\begin{array}{l} {\left|\alpha_{l,m}\right|}^{2}=\frac{\left|\Psi x_{l}\right|}{w_{l,m}^{1/2}\rho }\\ \Psi =B_{\left(t\right)}\Theta {\left(r_{l}{}_{\left(t\right)}\right)}^{-1}\;(t=0,1,\ldots ,N) \end{array}\right.$$

where $\rho =\sum_{m=1}^{N_{s}}w_{l,m}^{1/2}\left|\Psi x_{l}\right|+\sum_{N=N_{s}+1}^{N_{s}+NK}\frac{\left|\Psi x_{l}\right|}{{\left\|x_{l}\right\|}_{2}^{}}$;$w_{l,m}^{}=\frac{{\left\|a(f_{\mathrm{dm}},\psi_{\mathrm{sm}})\right\|}_{2}^{2}}{{\left\|x_{l}\right\|}_{2}^{2}}$. The solution of $R_{l}{}_{\left(t\right)}$ and $B_{\left(t\right)}$ is as follows [24]:

(32) $$\left\{\begin{array}{l} R_{l}{}_{\left(t\right)}=\sum_{m=1}^{N_{s}}\frac{\left|a{\left(f_{\mathrm{dm}},\psi_{\mathrm{sm}}\right)}^{H}x_{l}\right|}{{\left\|a(f_{\mathrm{dm}},\psi_{\mathrm{sm}})\right\|}_{2}^{2}}a(f_{\mathrm{dm}},\psi_{\mathrm{sm}}){\left(a(f_{\mathrm{dm}},\psi_{\mathrm{sm}})\right)}^{H}+\sigma_{n}I_{NK\times NK}\;(t=0)\\ B_{\left(t\right)}=\mathrm{diag}(\frac{\left|a{\left(f_{\mathrm{dm}},\psi_{\mathrm{sm}}\right)}^{H}x_{l}\right|}{{\left\|a(f_{\mathrm{dm}},\psi_{\mathrm{sm}})\right\|}_{2}^{2}})\;(t=0) \end{array}\right.$$

(33) $$\left\{\begin{array}{l} R_{l}{}_{\left(t\right)}=\sum_{m=1}^{N_{s}}{\left|\alpha_{l,m}\right|}_{\left(t-1\right)}^{2}a(f_{\mathrm{dm}},\psi_{\mathrm{sm}}){\left(a(f_{\mathrm{dm}},\psi_{\mathrm{sm}})\right)}^{H}+\sigma_{n}I_{NK\times NK}\;(t=1,2\ldots ,N)\\ B_{\left(t\right)}=\mathrm{diag}({\left|\alpha_{l,m}\right|}_{\left(t-1\right)}^{2})\;(t=1,2,\ldots ,N) \end{array}\right.$$

where Equation (32) is used to calculate $R_{l}{}_{\left(t\right)}$ and $B_{\left(t\right)}$ when t=0, and this is the first time the SPICE algorithm is used. Equation (33) is used when t=1,2,…,N to calculate $R_{l}{}_{\left(t\right)}$ and $B_{\left(t\right)}$ in the subsequent iteration cycle and in the Equation, ${\left|\alpha_{l,m}\right|}_{\left(t-1\right)}^{2}(t=1,2,\ldots ,N)$ is the ${\left|\alpha_{l,m}\right|}^{2}$ obtained from the previous cycle $R_{l}{}_{\left(t-1\right)}(t=1,2,\ldots ,N)$ and $B_{\left(t-1\right)}(t=1,2,\ldots ,N)$ are obtained from the cycle t.

Firstly, the constructed clutter dictionary is used to obtain $R_{l}{}_{\left(0\right)}$ and $B_{\left(0\right)}$ when the SPICE algorithm is first used according to Equation (32), and ${\left|\alpha_{l,m}\right|}_{\left(0\right)}^{2}$ is obtained according to Equation (31). Secondly, it is judged whether ${\left|\alpha_{l,m}\right|}_{\left(0\right)}^{2}$ meets the constraint condition of Equation (15). If the constraint condition is satisfied, ${\left|\alpha_{l,m}\right|}_{\left(0\right)}^{2}$ is the required ${\left|\alpha_{l,m}\right|}^{2}$. Otherwise, we put ${\left|\alpha_{l,m}\right|}_{\left(0\right)}^{2}$ into Equation (33) and iterate the cycle continuously until the constraint condition of Equation (15) is met at a certain iteration, then, ${\left|\alpha_{l,m}\right|}_{\left(t-1\right)}^{2}(t=1,\ldots ,N)$ that is obtained in this iteration is regarded as ${\left|\alpha_{l,m}\right|}^{2}$. Finally, we put ${\left|\alpha_{l,m}\right|}^{2}$ into Equation (14) to obtain the clutter covariance matrix $R_{l}^{}$.

**Figure 3:**
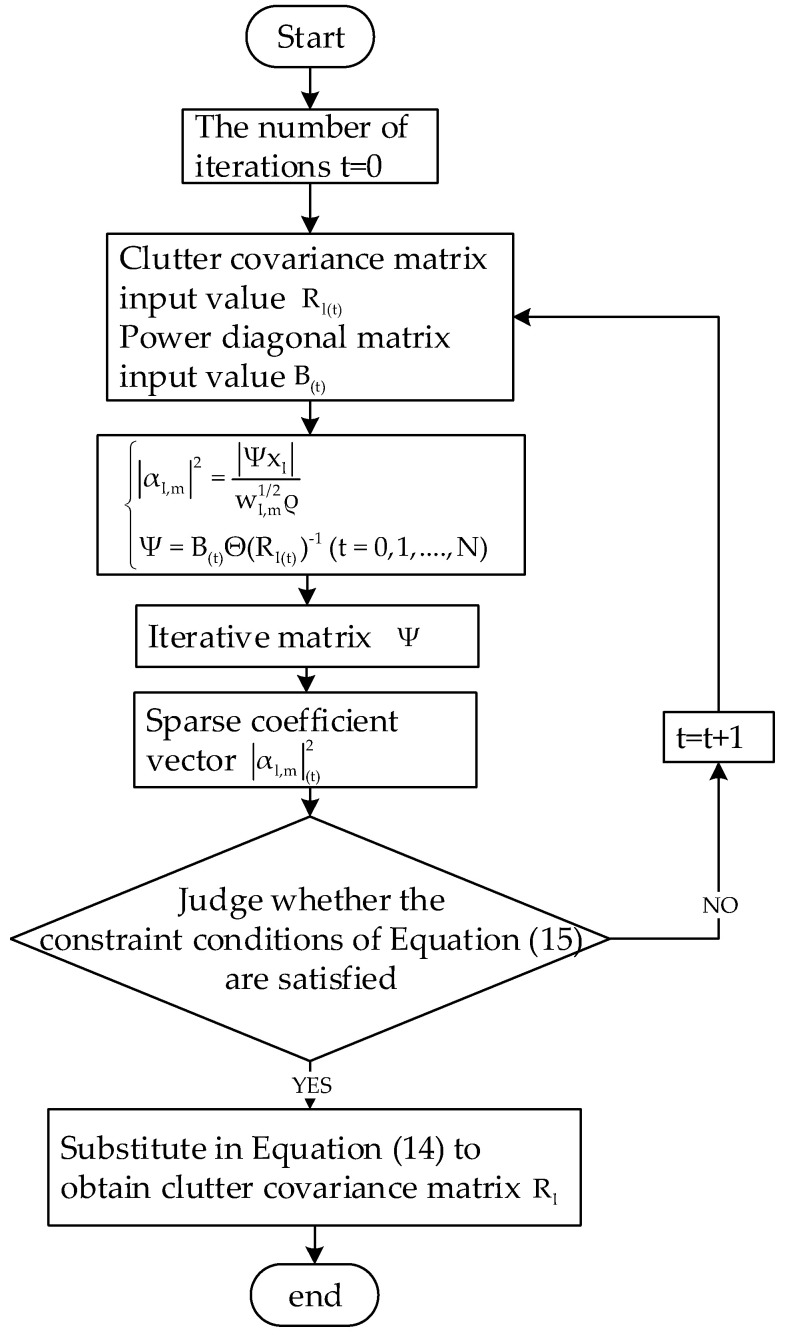
Flow diagram of clutter covariance matrix estimation based on SPICE algorithm.

The flow diagram of clutter covariance matrix estimation based on SPICE algorithm is shown in Figure 3 below.

### 3.2. Low-Altitude Windshear Wind Speed Estimation

After the clutter covariance matrix is obtained iteratively by the SPICE algorithm, the STAP processor is designed to suppress the ground clutter echo signal and estimate the wind speed. The optimal weight vector is solved by the following equation:

(34) $$\left\{\begin{array}{l} \min w_{l}^{H}R_{l}w_{l}^{}\\ s.t.w_{l}^{H}G_{l}=1 \end{array}\right.$$

where $G_{l}=v_{l}{\left(f_{d},\psi_{0}\right)}_{NK\times 1}$ is the extended low-altitude windshear space–time steering vector. According to Equation (34), the optimal weight vector is:

(35) $$w_{l}^{}=\mu R_{l}^{-1}G_{l}^{}$$

After the optimal weight vector $w_{l}^{}$ is used to process the radar echo signal, the ground clutter in the range unit $x_{l}$ that is to be detected is suppressed, and the normalized Doppler frequency is solved by the following equation:

(36) $${\hat{f}}_{l}=\underset{}{\mathrm{argmax}}w_{l}^{H}x_{l}$$

Then, the wind field target velocity estimation result of the lth range unit to be detected is:

(37) $${\hat{v}}_{l}=\frac{\lambda {\hat{f}}_{l}f_{r}}{4}$$

## 4. Process of the Method

**Figure 4:**
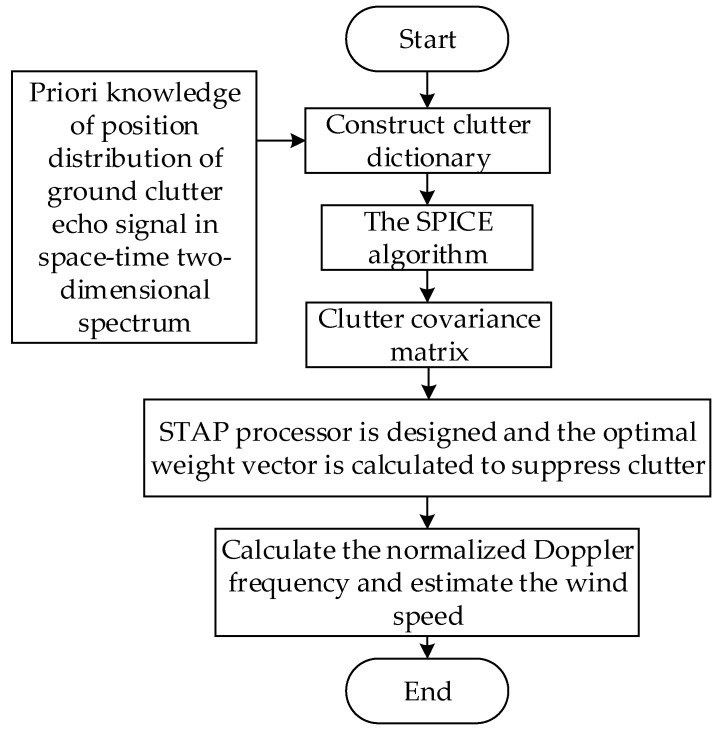
Flow diagram of low-altitude windshear wind speed estimation method based on KASPICE-STAP.

Figure 4 shows the flow diagram of the estimation method of low-altitude windshear wind speed based on KASPICE-STAP.

The method proposed in this paper is able to realize a low-altitude windshear wind speed estimation without IID training samples. The specific steps are as follows:

Step 1: Construct the corresponding clutter dictionary by using prior knowledge of the position distribution of ground clutter echo signals in space–time two-dimensional spectrum.

Step 2: Obtain the clutter covariance matrix iteratively using the SPICE algorithm.

Step 3: Design the STAP processor, calculate the optimal weight vector, and then suppress the ground clutter.

Step 4: After suppressing the ground clutter, the normalized Doppler frequency is obtained, and the wind speed for the detection range unit is estimated.

## 5. Results

In this paper, it is assumed that the low-altitude windshear wind field is located 8.5 km~16.5 km in front of the aircraft platform, the center of the low-altitude windshear wind field is located 12.5 away from the 60° direction of the aircraft platform, the main lobe of the antenna is aligned with the low-altitude windshear wind field, and the width of the low-altitude windshear wind field is 8 km. System simulation parameters are shown in Table 1.

**Figure 5:**
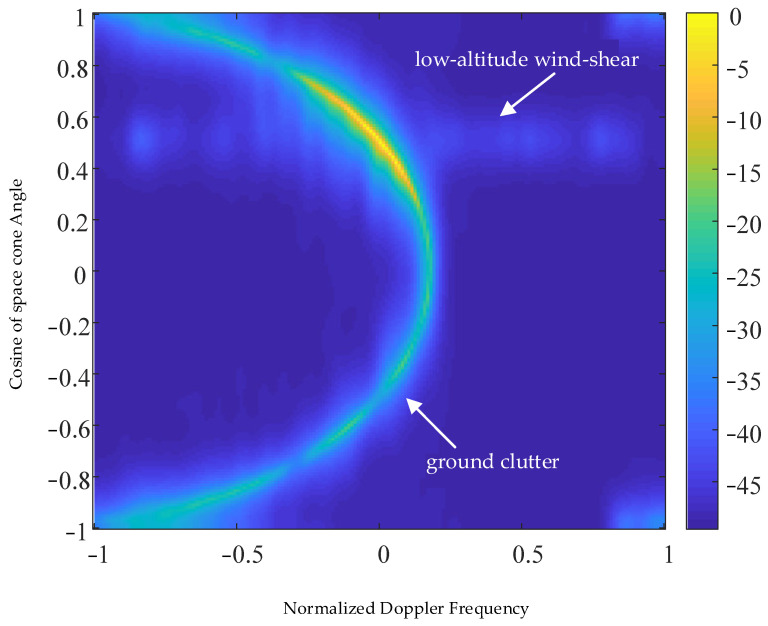
Two-dimensional space–time spectrum of radar echo signal.

Figure 5 shows the space–time two-dimensional spectrum of the radar echo signal of an airborne weather radar forward-looking array, wherein the ground clutter of the airborne forward-looking array has the space–time coupling characteristic, the ground clutter echo signal presents the semi-elliptical feature in the space–time two-dimensional spectrum, and the power spectrum of low-altitude windshear signal has a certain broadening in the normalized Doppler frequency domain and the spatial cone Angle cosine domain. It can be concluded from the analysis that the power of the ground clutter echo signal is much stronger than that of the low-altitude windshear signal, which makes the ground clutter echo signal completely cover up the normalized Doppler frequency information of the low-altitude windshear echo signal, which is an important factor causing the inaccurate performance of the low-altitude windshear estimation.

**Figure 6:**
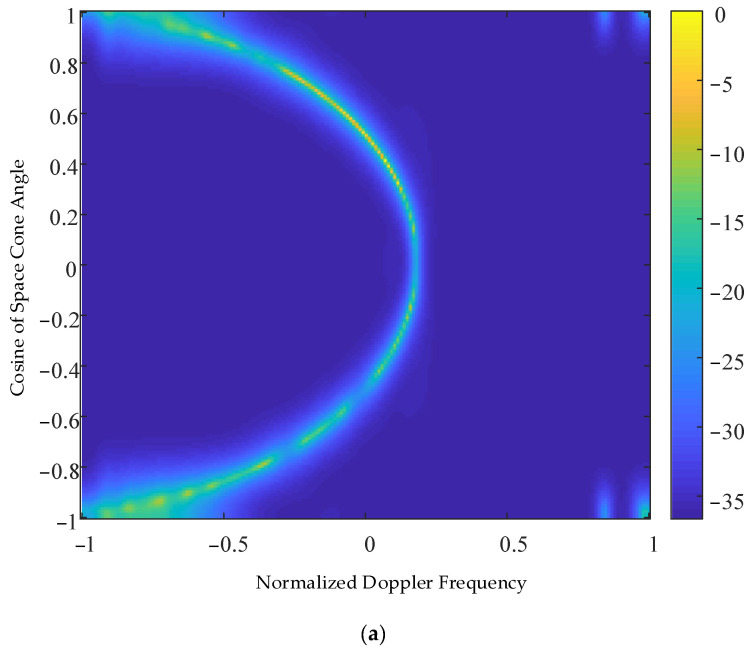

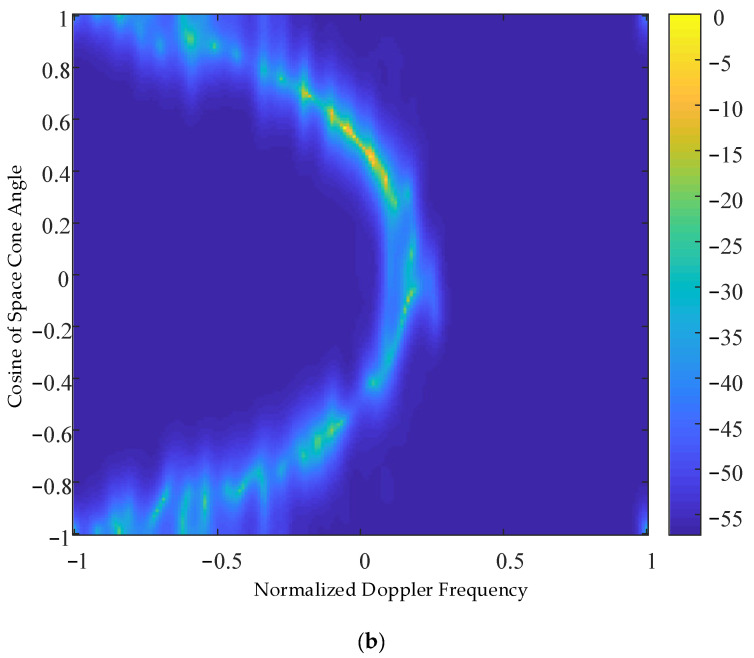
Space–time two-dimensional spectrum of ground clutter recovered by KASPICE-STAP method and DDD-SR-STAP method. (**a**) KASPICE-STAP method. (**b**) DDD-SR-STAP method.

Figure 6a,b shows the space–time two-dimensional spectrum of the ground clutter obtained using the KASPICE-STAP method and DDD-SR-STAP method for the sparse recovery of the detection range units, respectively. As can be seen from Figure 6, although the DDD-SR-STAP method recovers the space–time two-dimensional spectrum of the ground clutter echo signal, it also presents semi-elliptical distribution characteristics, the space–time spectrum of ground clutter echo signal has a serious discontinuous distribution, and the recovery effect of the space–time two-dimensional spectrum of ground clutter echo signal is very poor. The space–time two-dimensional spectrum of the ground clutter echo signal obtained by the sparse recovery method in this paper is more accurate, and the semi elliptical distribution characteristics and the main lobe of the ground clutter echo signals can be clearly seen, which is more consistent with the space–time two-dimensional spectrum of the ground clutter echo signal in Figure 5 radar echo signal, showing the ultra-high resolution performance advantages of the sparse recovery technology.

**Figure 7:**
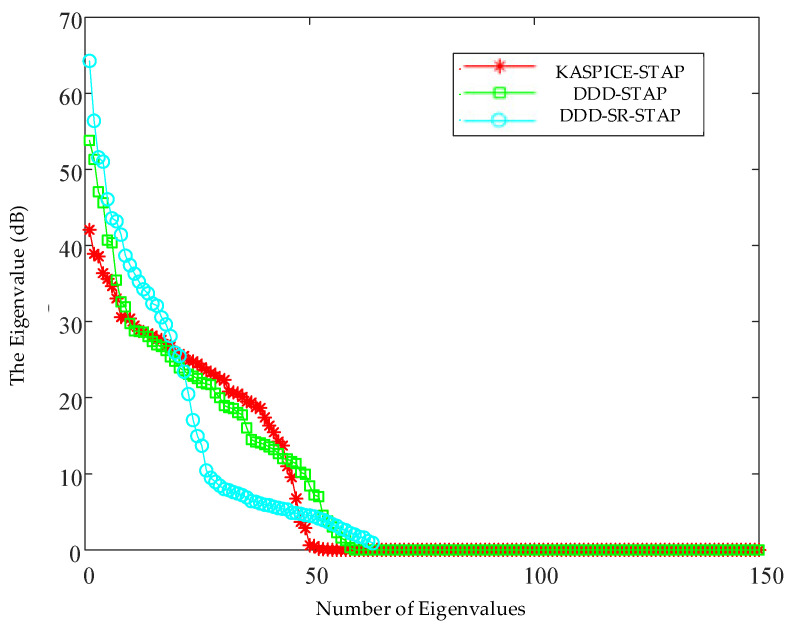
Clutter characteristic spectrum.

Figure 7 shows the clutter characteristic spectra of the clutter covariance matrix estimated by the KASPICE-STAP method, the DDD-STAP method, and the DDD-SR-STAP method. It can be seen from Figure 7 that the number of large eigenvalues of the clutter covariance matrix estimated by the KASPICE-STAP method is smaller than that of the other two methods, that is, the clutter degree of freedom is low, so the clutter covariance matrix estimated by the KASPICE-STAP method is more accurate.

**Figure 8:**
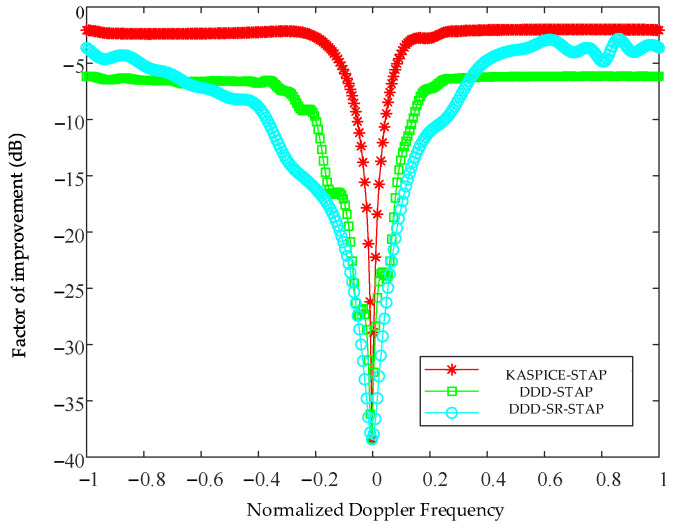
Factor of improvement curve of No. 60 range unit.

Figure 8 shows the factor of improvement curves of the KASPICE-STAP method, the DDD-STAP method, and the DDD-SR-STAP method. Taking the No. 60 range unit as an example, it can be seen from Figure 8 that compared with the other two methods, the KASPICE-STAP method has a deeper and narrower notch in the main clutter area, which is able to suppress the clutter echo signals more effectively.

**Figure 9:**
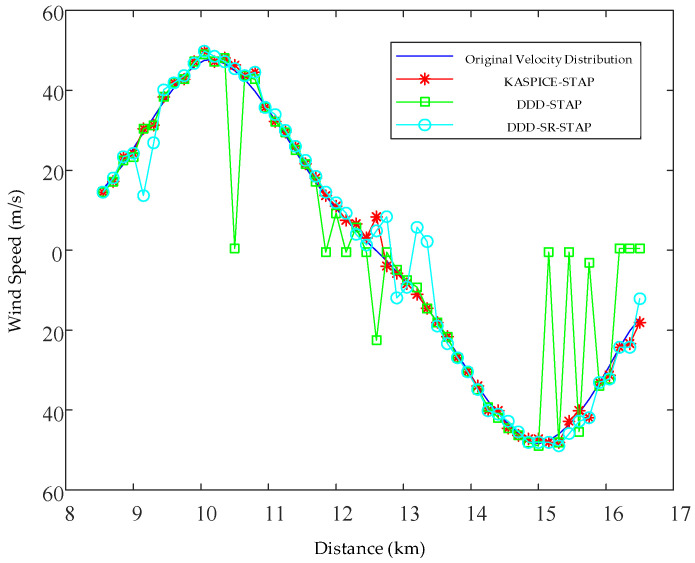
Comparison of low-altitude windshear wind speed estimation.

Figure 9 shows the comparison of the low-altitude windshear wind speed estimations by the KASPICE-STAP method, the DDD-STAP method, and the DDD-SR-STAP method. As can be seen from Figure 9, the DDD-STAP method cannot accurately estimate the low-altitude windshear wind speed due to the loss of degrees of freedom in the sliding window and the limitation of the degrees of freedom in the space and time domain. The DDD-SR-STAP method is affected by only one single range unit data that are to be detected, and the accuracy of low-altitude windshear wind speed estimation is not high. The KASPICE-STAP method can obtain better wind speed estimation results with higher accuracy. At the same time, within the range of the 8.5 km–16.5 km low-altitude windshear wind field, the wind speed of the low-altitude windshear wind field shows an inverse “S” shape with distance.

Table 2 shows the comparison of root mean square error of low-altitude windshear wind speed estimation by the KASPICE-STAP method, the DDD-STAP method, and the DDD-SR-STAP method. It can be seen from Table 2 that the root mean square error of low-altitude windshear wind speed estimation by the DDD-STAP method and the DDD-SR-STAP method is larger than that which was achieved by the KASPICE-STAP method. Therefore, the KASPICE-STAP method can achieve accurate estimation results of low-altitude windshear wind speed.

## 6. Conclusions

When one is estimating low-altitude windshear wind speed, being faced with no IID training samples is a common situation. The traditional STAP method is unable to accurately estimate the clutter covariance matrix and suppress the clutter, which severely degrades the performance of low-altitude windshear wind speed estimation. In this paper, a low-altitude wind-speed estimation method based on KASPICE-STAP was designed. This method was applied to airborne weather radar, and it can be used for the clutter suppression and parameter estimation of distributed targets without any IID training samples, and it shows a good performance when there is only a single range unit that is to be detected. It uses prior knowledge of the ground clutter echo signals, fully excavated the sparsity of the space–time two-dimensional spectrum of the range unit that is to be detected to construct the clutter dictionary, and then, it uses the SPICE algorithm to iteratively obtain the clutter covariance matrix. Compared with the traditional DDD-STAP method, this method obtains a more accurate clutter covariance matrix due to its excellent performance. Finally, the STAP processor was designed, and the corresponding optimal weight vector was calculated, the normalized Doppler frequency was proposed from the signal after eliminating the ground clutter afterwards, and ultimately, the wind speed was estimated. The simulation results showed that the KASPICE-STAP method can still effectively and accurately estimate the low-altitude windshear speed without IID training samples. At present, the limitation of the method is that the clutter dictionary is traversed in each iteration of the SPICE algorithm, which has a high computational complexity and leads to a long operation time. This will be our future research direction.

## Figures and Tables

**Table 1:** System simulation parameters.

Parameter	Value	Parameter	Value
Aircraft platform height (m)	600	Number of array elements	8
Aircraft platform speed (m/s)	75	Number of coherent pulse	32
Radar wavelength (m)	0.032	Angle of main lobe (°)	(60,0)
Pulse repetition frequency (Hz)	7000	Range resolution (m)	150
Signal-to-noise ratio (dB)	5	Clutter-to-noise ratio (dB)	40

**Table 2:** Comparison of root mean square error of low-altitude windshear wind speed estimation.

Wind Speed Estimation Method	Root Mean Square Error/(m/s)
DDD-STAP	13.2187
DDD-SR-STAP	4.7335
KASPICE-STAP	1.8692

## Data Availability

Not applicable.
